# An empirical analysis of social public resources digital sharing system: Dissipative structure theory

**DOI:** 10.1371/journal.pone.0271300

**Published:** 2022-07-21

**Authors:** Shengzhu Li, Fan Jiang

**Affiliations:** School of Economics and Management, Chongqing University of Posts and Telecommunications, Chongqing, China; Universidad Nacional Autonoma de Nicaragua Leon, NICARAGUA

## Abstract

The development of the social public resource digital sharing system (SPRDSS) has been accelerated with the evolution of digital information and communication technologies (ICTs). This paper analyzes the dissipative structure features and formation process of SPRDSS in China. Combined with the Brusselator model and its transformation, this paper empirically analyzes the dissipative structure of SPRDSS using panel data collected from 30 Chinese provinces (excluding Tibet, Hong Kong, Macao, and Taiwan) from 2015 to 2019. The results show that the SPRDSS in China has pre-conditions to form a dissipative structure. At present, the SPRDSSs in most Chinese provinces have not yet formed the dissipative structure, but they are gradually evolving into it. The global orderliness of the sharing system is greater in eastern China than in central China and greater in central China than in western China. The potential for improving global orderliness is greater in western China than in central China and is greater in central China than in eastern China. Therefore, proper policies and measures should be adopted to accelerate the construction of SPRDSS based on the evolution of dissipative structure and to promote the sustainable development of the digital sharing economy.

## Introduction

With the rapid development of modern information and communication technologies (ICTs) such as big data, the Internet of Things (IoT), and 5G, people in and across diverse and spatially distributed groups can easily and quickly gain social public resources (SPR) through digital sharing with few constraints of time, place, and communication mode [1]. During the COVID-19 epidemic, various countries and areas have established targeted epidemic prevention and control systems based on travel itineraries and epidemic information sharing. This has improved the efficiency of epidemic prevention and control and reduced the risk of COVID-19 infection [2, 3]. Relying on digital media and integrating public resources such as healthcare, education, and public services, governments can utilize public service platforms such as telemedicine, online education, and e-government, to effectively solve the public’s problems, and address the negative impact of COVID-19 [3]. For example, in Nicaragua, digital platforms were used to share educational resources for learning in a virtual environment, which helped to avoid greater dropout rates and inequality, as well as the economic crises [4]. Therefore, the digital sharing of SPR using ICTs, can improve and enhance the balanced allocation and efficient utilization of public resources, as well as effectively respond to public emergencies. Digital sharing can promote the sustainable and healthy development of sharing economy, and better meet the material and spiritual needs of the public. Such digital sharing activities involve multiple subjects and elements, such as e-government departments and business organizations that provide (receive) digital sharing services, as well as material and immaterial public resources that can be digitally shared [5]. The sharing behavior between relevant subjects and the flow of public resources constitute the social public resources digital sharing system (SPRDSS). It is a complex open system with multiple subjects, multilateral relationships, and multi-level structures [6].

ICTs can promote the digital sharing of public resources, enhance social public services, and reduce inequalities [7, 8]. In education, the cloud platform sharing digital educational fabrication resources could be constructed by leveraging cloud and IoT technologies and by providing remote access to distributed expensive fabrication resources over the internet. It can promote the interaction between education and manufacturing R&D [9]. Similarly, in healthcare, a cross-jurisdictional resource-sharing service model in local health departments can provide at least as many mandated and more nonmandated services with greater quality [10]. However, public resource sharing is affected by the benefits, costs, and risks of sharing [11]. Pereira and Fong proposed the SEPD model as an access control model to address the problems of policy administration and trust establishment in public resource sharing [12]. Palgan et al. proposed five sharing economy governance strategies to enable burden-sharing, promote public benefits, and pursue the common good [13, 14]. Many scholars have explored the models and system architecture of digital sharing of public resources, as well as its realization paths [15, 16]. However, what are the evolutionary states of such a complex open system that co-evolves with the external environment through the exchange of matter, energy, and information? What is the impact of external inputs on the system’s evolution? A few studies have discussed these issues.

Dissipative structure theory has been applied to study the evolution of complex systems, such as manufacturing systems and stock market structures [17, 18]. As an important stage in the evolution of complex systems, a dissipative structure is a stable structure with self-organizing characteristics formed by an open, nonlinear system [19]. Such a system can continuously exchange material and energy with the external environment and evolve from disorder to order, low level to high level through the interaction of system elements to adapt to changes in the external environment. According to Prigogine’s elaboration of dissipative structure, a system may form a dissipative structure under the following conditions: (1) openness. An open system continuously exchanges material and energy with the external environment, regulating the entropy of the system, thus forming a dissipative structure; (2) fluctuation. The reactions that occur with the fluctuations cause the system to form an overall ordered structure; (3) far from equilibrium. Systems far from equilibrium can form great fluctuations, evolving toward an ordered state; (4) nonlinearity. The complex nonlinear interactions within the system elements produce a new stable state [19, 20]. Complex systems that meet the above four conditions have adaptive (self-organizing) characteristics. It can form a dissipative structure to achieve the evolution from disorder to order, low level to high level, and simple to complex. Song and Guo introduced Bayesian analysis to analyze the dissipative structure change of the Chinese stock market from the perspective of probability theory [18]. Deng et al. used the Brusselator model to develop the structure of the building energy service industry system and constructed a model to verify the system by the entropy method [21]. Li et al. verified four evolution mechanisms of internet public opinion diffusion based on four factors, the public health emergency, netizen, network media, and government, to improve the governance ability of the opinion [22].

In summary, existing literature on social resource sharing focuses more on the mechanisms and models of sharing and less on this sharing system as a complex system. Recent research rarely used the theories and methods of complexity science to examine the structural characteristics and evolutionary laws of the sharing system. Therefore, taking China’s SPRDSS as an example, this paper analyzes the dissipative structure features of SPRDSS based on the dissipative structure theory. Then we conduct an empirical analysis to verify the dynamic evolutionary law of SPRDSS, combined with the Brusselator model and its transformation. This is important to promote the digital sharing of SPR and maintain sustainable socio-economic development.

## Social public resources digital sharing system

### Internal structure

**Fig 1:**
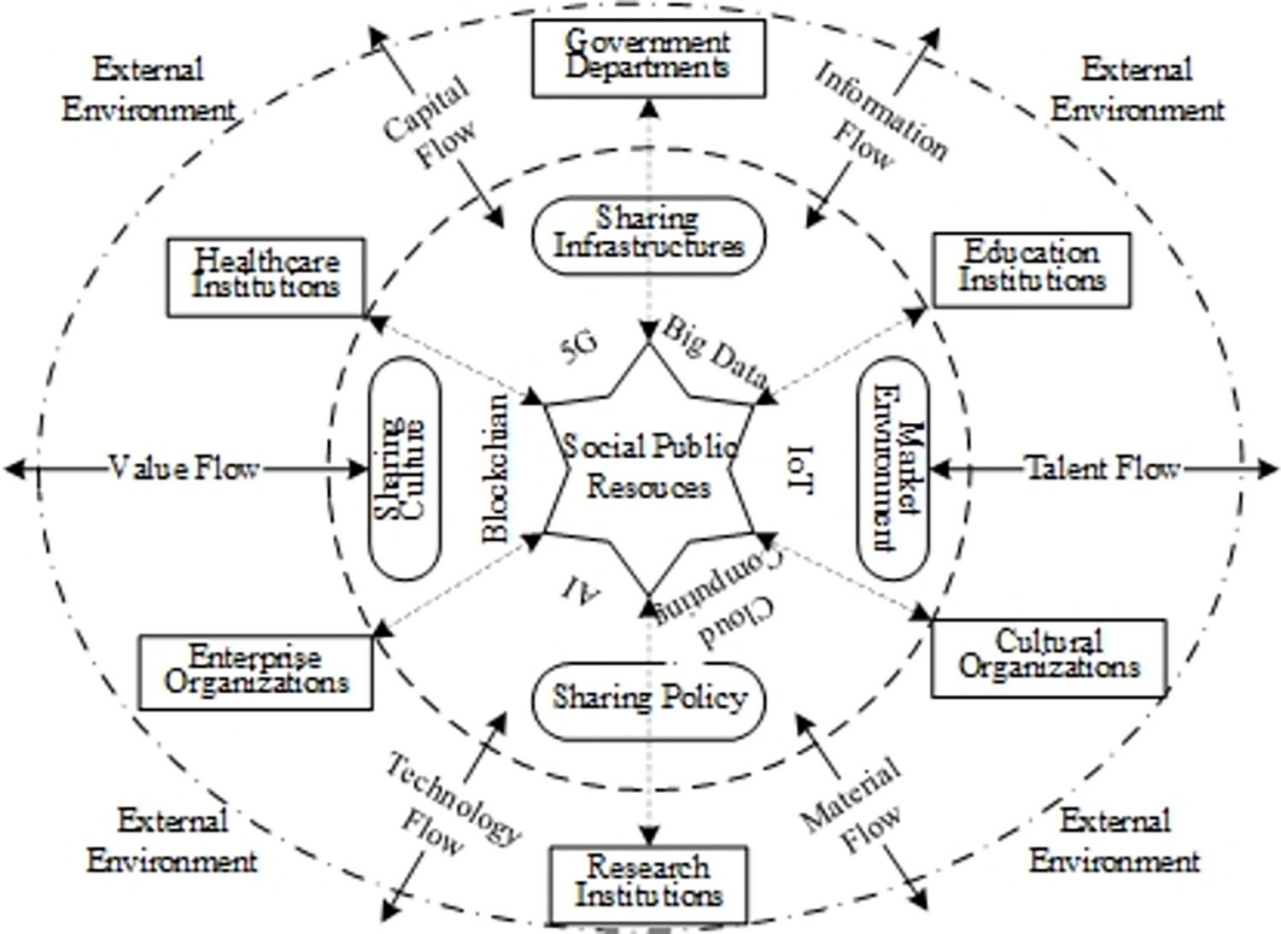
The structure of the digital sharing system of SPR.

To clarify the generation mechanism and formation process of the dissipative structure of SPRDSS, it is necessary to define the internal elements and structure of the system. Based on relevant literature [23], this paper assumes that SPRDSS mainly consists of three major components: sharing resources, environment, and subjects, as shown in Fig 1.

The first layer is the resources, which refers to the shared resource objects of digital sharing of SPR, including tangible and intangible resources that can be shared digitally [1]. These resources mainly refer to public resources such as educational resources, scientific research data, equipment and technology resources, cultural resources and services, healthcare information and technology, and social security and public services that can be provided through digital platforms. The uneven distribution of public resources motivates resource subjects to obtain the right to use these resources through sharing. Hence, the creation and transfer of public resources’ value and the maximization of social benefits can be realized. Through digital sharing, it is possible to increase the circulation rate and utilization rate of SPR and avoid idleness and waste of resources [1].

The second layer is the environment, which refers to the regional sharing environment of the digital sharing of SPR [24]. It mainly consists of four parts: (1) the sharing policy system such as policy institution and financial regime promulgated by local governments in the sharing area for digital sharing of SPR; (2) the regional market environment such as stable or chaotic market and investment environment; (3) the sharing infrastructures related to sharing activities in the system, such as network facilities, sharing platforms; and (4) the positive or negative regional sharing culture, which is mainly reflected in the degree of support and the intensity of willingness to participate in SPRDSS among various subjects within the system [25].

The third layer is the subjects, which refers to the resource supply and demand subjects of the digital sharing of SPR. It mainly includes e-government departments, educational institutions, research institutions, health institutions, cultural organizations, and firms that can provide (receive) digital sharing services. The subjects are both resource suppliers and demanders of resources in the system, pursuing the efficient utilization of resources and the maximization of their benefits as well as social benefits. For example, through the digital health platform, different healthcare institutions can store and share medical and health resources such as case data and medical equipment information while data security and patient privacy are ensured. Hence, information sharing, medical collaboration, and assistance can be easily carried out between healthcare professionals and institutions, which will greatly contribute to healthcare treatment, pharmaceutical R&D, and medical technology innovation [26].

SPRDSS takes the sharing environment as a carrier, and comprehensively applies information network technologies, which aims to allocate public resources equally, and to use them more efficiently. SPRDSS improves urban-rural social welfare benefits by circulating and renewing internal public resources with the mutual constraints and cooperation of internal sharing subjects [27]. The system continuously adjusts its internal structure and status through material and capital flow, talent and technology exchange, and information and value interchange with the external environment. It carries out metabolism to maintain its integrity and stability and promote further replacement and evolution of the system.

### Dissipative structure features

SPRDSS is an open and complex self-organizing system with typical features of a dissipative structure.

#### (1) Openness

The openness of SPRDSS, which is composed of internal and external openness, is the prerequisite for the survival and development of the system. Internal openness refers to the spatio-temporal circulation and exchange of information, capital, materials, and other resources using ICTs between sharing subjects, and between sharing subjects and the environment. External openness refers to the circulation and exchange of financial capital, technical information, talent, and other resources between the sharing system and the external environment through financial transfer payment, talent introduction, technical exchange, and other ways. While maintaining openness, the digital sharing system is continuously updated alternatively to realize the evolution and upgrade.

#### (2) Fluctuation

Fluctuation refers to the situation where the system is affected by the dynamic changes of multiple internal and external factors, resulting in random fluctuations. The random fluctuations will destabilize the system structure. Through nonlinear reaction, a great fluctuation will be formed, which will trigger the system mutation and promote the system to produce a macro-ordered structure. The fluctuations of the system are common, e.g., the change in sharing policy and regulations, innovation, and iteration of digital technology. Under certain conditions, a small fluctuation may develop into a great fluctuation, triggering system mutations, forming bifurcations, and prompting the system to evolve in different directions. Properly handling the linear relationship of mutation points will enable the system to realize the transformation from disorder to order and to form a new dissipative structure.

#### (3) Unbalance

The unbalance of SPRDSS refers to the state where the system is far from equilibrium. Due to the complex operation of the system, there are great differences in the sharing ability, resource status, and technical level of each subject. Hence, obvious nonlinear effects arise within the sharing system, which can lead to the formation of an orderly system [28]. In addition, equilibrium refers to the stable and orderly digital sharing of SPR between the sharing subjects by ICTs. It is often broken due to technological updates in the external environment, changes in government policies and regulations, changes in the quantity and sharing willingness of sharing subjects within the system, and other random changes. Consequently, the sharing system is in the nonlinear zone far from equilibrium. The system is chaotic when it is far from equilibrium, forming a macro-disorder and micro-order structure. The system will not reach a higher level of the evolutionary stage until a new equilibrium is sought.

#### (4) Non-linearity

The nonlinearity of SPRDSS refers to the mutual promotion and limitation between the system elements, which would form a complex positive or negative feedback. The effects of the interactions are not only simple sums of the parts, but also include synergistic effects between the system elements. Due to such nonlinear influence relation, the system can achieve a qualitative breakthrough under the influence of external inputs and each element. It can develop from disorder to order, from low order to high order.

### Dissipative structure formation process

As an open and complex system, SPRDSS gradually increases its internal negative entropy when capital, information, talent, technology, and other materials and energy are continuously exchanged with the external environment. The inflow of negative entropy can not only offset the positive entropy generated by the existing technology and resources in the system but also reduce the total entropy of the system. This will cause system mutation, and promote the transition from the disordered original structure to the stable non-equilibrium new structure.

**Fig 2:**
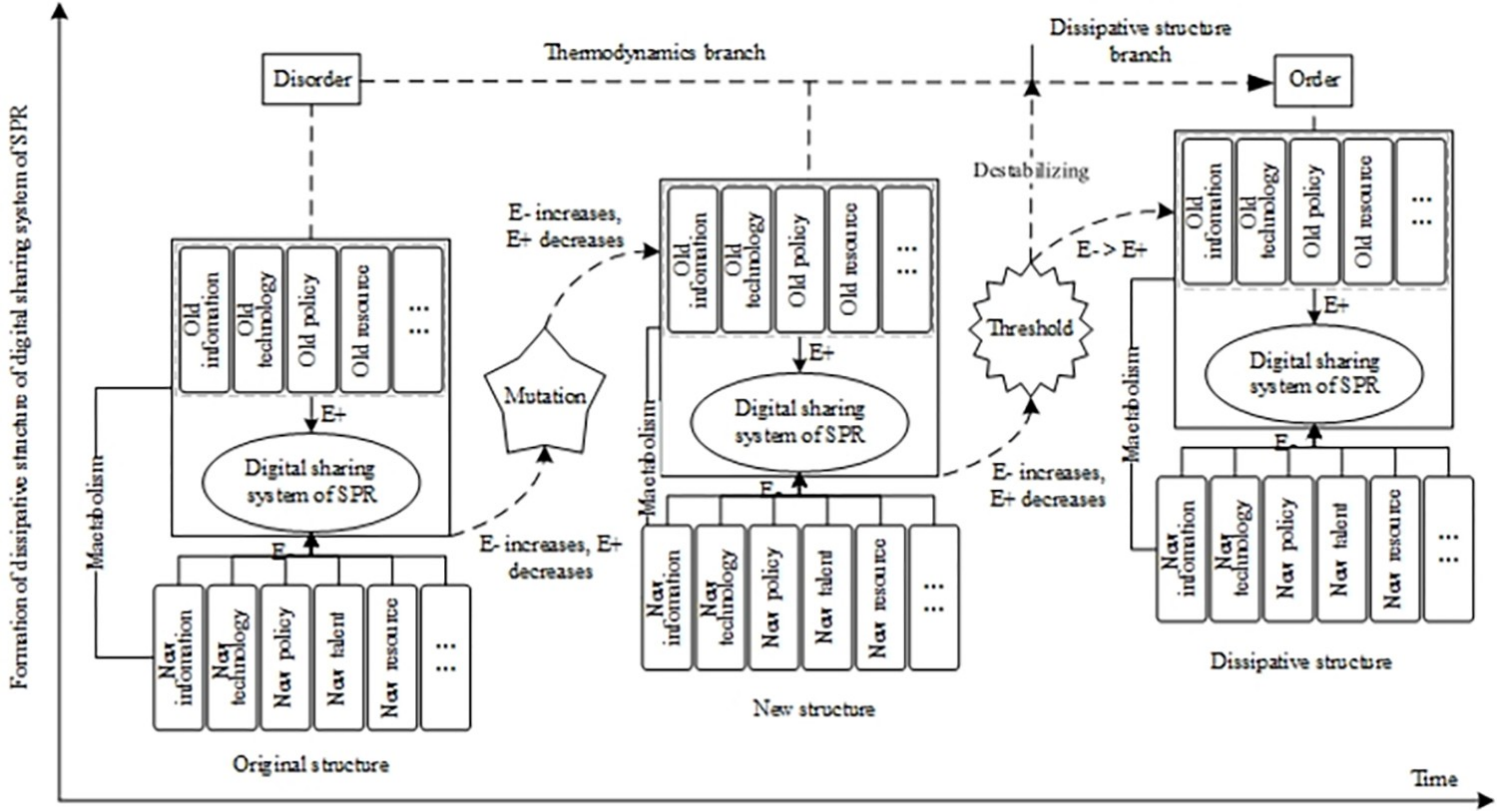
The formation process of the dissipative structure of digital sharing of SPR. *E- stands for negative entropy and E+ for positive entropy.

When the total entropy of the system reaches a certain threshold, the great fluctuation triggered by random fluctuations makes the thermodynamic branches of the system unstable, and the system spontaneously changes its internal structure. After undergoing mutation and bifurcation, the system gradually reaches the macro-ordered dissipative structure state from the chaotic state. The specific formation process is shown in Fig 2.

The dissipative structure of SPRDSS is a macro-ordered and relatively stable state formed on its original structure. It is an ideal structure of stable and orderly development of the sharing system with self-organizing features in the spatial dimension. Therefore, the original system structure of SPRDSS is the foundation of its dissipative structure, while the dissipative structure is the specific manifestation of the continuous evolution and development of the system in the time dimension.

## Model and data

### The Brusselator model

According to the above analysis, the dissipative structure is a stable and ordered state achieved by the system’s mutation after the system becomes destabilized due to some random fluctuations and nonlinearity influence. In this paper, the Brusselator model and its transformation are used to analyze the generation mechanism and formation conditions of the dissipative structure of SPRDSS.

#### Model description

The Brusselator model consists of a single nonlinear (three molecules) reaction that describes the evolutionary process of two chemical intermediates *X* and *Y* [29]. The model is a mathematical model, which can be used to simulate the self-organizing phenomenon of a given system and identify whether the system achieves a dissipative structure accurately and rationally [30]. It mainly includes four chemical reaction formulas, as follows

$$A\overset{k_{1}}{\to }X$$ (1)

$$B+X\overset{k_{2}}{\to }Y+D$$ (2)

$$2X+Y\overset{k_{3}}{\to }3X$$ (3)

$$X\overset{k_{4}}{\to }E$$ (4)

In the above reaction formulas, *A* and *B* are the initial reactants, which can be replenished by the external environment, and their concentrations remain the same during the reaction. *D* and *E* are the reaction products, which are taken away once they are produced. *X* and *Y* are intermediates whose concentrations may vary with the reaction time. Due to autocatalytic reaction (3), the concentration of *X* in the reaction gradually increases. The change of reactant concentration can be metaphorically described as the interaction of system elements. For example, reaction (3) could reflect the synergistic relationship between *X* and *Y*. *k*_1_, *k*_2_, *k*_3_, and *k*_4_ are the catalysts, which directly affect the overall chemical reaction rate.

From a system operating perspective, the digital sharing of SPR mainly goes through three stages.

Stage 1. SPR is transported by ICTs from the regional sharing environment to the sharing subject, which leads to a series of competitive-cooperative actions of the subjects;
Stage 2. The complex interactions of the sharing subjects lead to the effective allocation and flow of public resources. And then SPRDSS generates socio-economic benefits such as balanced high-quality regional development;
Stage 3. The resource sharing benefits are reinvested into the regional sharing environment, and continue to participate in this process and improve the sharing level and capacity of the subjects.

Stage 1 is the basis of stages 2 and 3, stage 2 is the core process of the digital sharing of SPR, and stage 3 has a certain feedback effect on stages 1 and 2. This paper finds that this process is similar to the chemical reaction process in the Brusselator model. Based on the above analysis and combined with relevant knowledge of SPRDSS, the following economic transformation of the Brusselator model is made, as shown in Table 1.

**Table 1:** The transformations of the Brusselator model.

Elements	Transformations	Elements	Transformations
*A*	Financial input of digital sharing of SPR	*B*	The environment of digital sharing of SPR
*X*	Digital sharing level of the system	*Y*	The balanced degree of regional socio-economic development
*D*	Digital sharing benefits of SPRDSS	*E*	Digital sharing system replacement
*k*_1_	Reciprocal of the lag time that sharing inputs improve sharing level	*k*_2_	Reciprocal of the lag time to generate benefits of digital sharing of SPR
*k*_3_	Reciprocal of the lag time that the balanced degree of regional socio-economic development improves sharing level	*k*_4_	Reciprocal of the lag time for the evolution of digital sharing system replacement

In Table 1, *A* and *B* respectively represent the financial inputs and environmental support of digital sharing of SPR, which are kept constant because of the continuous inflow of financial capital and other external elements. *D* represents the digital sharing benefits of SPRDSS, which is mainly used to compensate for the costs of resources digital sharing (e.g., maintenance costs of the sharing platform, depreciation of the infrastructure used for sharing, etc.) and the opportunity costs of sharing subjects. It has no direct impact on the digital sharing of SPR in the current period and will be taken away once it is generated. *E* indicates the evolution and replacement of SPRDSS, the change of new and old public resources, and the elimination and upgrading of sharing policies, talent, and technologies within the system. *X* indicates the sharing level of the system (e.g., sharing willingness of system subjects, the scale of shared resources, etc.). *Y* indicates the balanced degree of regional socio-economic development. *k*_1_, *k*_2_, *k*_3_, and *k*_4_ correspond to the "reaction rate".

Reaction 1: $A\overset{k_{1}}{\to }X$. It indicates that SPRDSS triggers a series of competitive-cooperative actions of the internal subjects after receiving financial capital for the creation and sharing of SPR. In this way, the sharing level of the system can be improved, that is, the sharing willingness of the subject and the quantity and type of the shared resources can be enhanced [14]. This process shows that sharing inputs can improve the sharing level.
Reaction 2: $B+X\overset{k_{2}}{\to }Y+D$. It means that the sharing subjects, after the sharing level is improved, actively share their resources through the digital sharing environment to allocate public resources equally. It can improve the balanced degree of regional socio-economic development [24], and obtain the benefits of resource sharing.
Reaction 3: $2X+Y\overset{k_{3}}{\to }3X$. It is the autocatalysis of the system sharing level, representing that the more balanced degree of regional socio-economic development, the higher the system sharing level. In reactions 2 and 3, *X* and *Y* reflect that the balanced degree of regional socio-economic development can positively influence the sharing willingness of system subjects and the scale of shared resources [31], which can further improve the sharing benefits.
Reaction 4: $X\overset{k_{4}}{\to }E$. It represents the evolution and replacement of SPRDSS, which is mainly reflected in the flow, consumption, and change of new and old public resources, the transformation of sharing subjects, and the elimination and upgrading of sharing policies, talent, and technologies. This process will change the sharing willingness of system subjects and reduce the resource sharing scale, which will lower the system sharing level.

#### Model analysis

According to the law of mass action and the original chemical reaction formula, the translated Brusselator reaction diffusion kinetic equation [30] is

$$\{\begin{array}{l} \frac{dx}{dt}=k_{1}A-k_{2}Bx+k_{3}x^{2}y-k_{4}x\\ \frac{dy}{dt}=k_{2}Bx-k_{3}x^{2}y \end{array}$$ (5)

In which *A*, *B*, *x*, and *y* respectively represent the dimensionless processing results of the financial inputs of SPRDSS, environmental support, the system sharing level, and the balanced degree of regional socio-economic development. There are two ways to determine the reaction rates (*k*_1_, *k*_2_, *k*_3_, and *k*_4_). One is to establish the kinetic equation in the dimensionless form, assuming that the reaction rates are 1 or 2 [30], and the other is to make a specific analysis of the transformation combined with the real situation and to determine the values of *k*_1_, *k*_2_, *k*_3_, and *k*_4_ [32]. To increase the accuracy, objectivity, and rationality of the Brusselator model, the second method is selected. And *k*_1_ = 1/2, *k*_2_ = 1, *k*_3_ = 1/5, and *k*_4_ = 1/4 are set according to related literature [33–36]. Furthermore, the specific values of *k*_1_, *k*_2_, *k*_3_, and *k*_4_ are not substituted into them in the next analysis to ensure the generalizability of the model analysis.

The set of stationary state equations of the kinetic Eq (5) is as follows.


$$\{\begin{array}{l} \frac{dx}{dt}=k_{1}A-k_{2}Bx+k_{3}x^{2}y-k_{4}x=0\\ \frac{dy}{dt}=k_{2}Bx-k_{3}x^{2}y=0 \end{array}$$ (6)

Solve it to obtain the stationary state solution, $x_{0}=\frac{k_{1}}{k_{4}}A$, and $y_{0}=\frac{k_{2}k_{4}}{k_{1}k_{3}}\frac{A}{B}$. Here, *x* and *y* do not reflect the orderliness degrees of *X* and *Y*. As can be seen from the above, the dissipative structure of SPRDSS is only possible when the system is unstable. Therefore, the stability analysis of the stationary state solution is needed to find the critical conditions for the system to evolve from instability to dissipative structure.

Let

$$x=x_{0}+\Delta x\;y=y_{0}+\Delta y$$

$$\{\begin{array}{l} F(x,y)=k_{1}A-k_{2}Bx+k_{3}x^{2}y-k_{4}x\\ G(x,y)=k_{2}Bx-k_{3}x^{2}y \end{array}$$ (7)

According to Eq (7), the Jacobian matrix of the stationary state solution (*x*_0_, *y*_0_) is

$$J(x_{0},y_{0})={\left[\begin{array}{c} \frac{\partial F}{\partial x}\;\frac{\partial F}{\partial y}\\ \frac{\partial G}{\partial x}\;\frac{\partial G}{\partial y} \end{array}\right]}_{\left(x_{0},y_{0}\right)}=[\begin{array}{cc} k_{2}B-k_{4} & \frac{k_{1}^{2}k_{3}}{k_{4}^{2}}A^{2}\\ -k_{2}B & -\frac{k_{1}^{2}k_{3}}{k_{4}^{2}}A^{2} \end{array}]$$ (8)

Thus, the linearized matrix form of the Eq (7) can be expressed as

$$\left[\begin{array}{c} F(\Delta x,\Delta y)\\ G(\Delta x,\Delta y) \end{array}\right]=[\begin{array}{cc} k_{2}B-k_{4} & \frac{k_{1}^{2}k_{3}}{k_{4}^{2}}A^{2}\\ -k_{2}B & -\frac{k_{1}^{2}k_{3}}{k_{4}^{2}}A^{2} \end{array}]\left[\begin{array}{c} \Delta x\\ \Delta y \end{array}\right]$$ (9)

The characteristic equation of the linearized form of differential Eq (9) is $\lambda^{2}-\left(k_{2}B-k_{4}-\frac{k_{1}^{2}k_{3}}{k_{4}^{2}}A^{2}\right)\;\lambda +\frac{k_{1}^{2}k_{3}}{k_{4}}A^{2}=0$. According to the Hurwitz criterion, the system is stationary when all coefficients of the characteristic equation are positive [37], and it cannot form the dissipative structure. Accordingly, the conditions for determining the dissipative structure of SPRDSS are

1. $k_{2}k_{4}^{2}B<k_{4}^{3}+k_{1}^{2}k_{3}A^{2}\cdots \cdots \cdots$The stationary state solution is in a stationary state and the system cannot form a dissipative structure.
2. $k_{2}k_{4}^{2}B=k_{4}^{3}+k_{1}^{2}k_{3}A^{2}\cdots \cdots \cdots$The stationary state solution is a stable point.
3. $k_{2}k_{4}^{2}B>k_{4}^{3}+k_{1}^{2}k_{3}A^{2}\cdots \cdots \cdots$The stationary state solution is in a non-stationary state and the system can form a dissipative structure.

**Fig 3:**
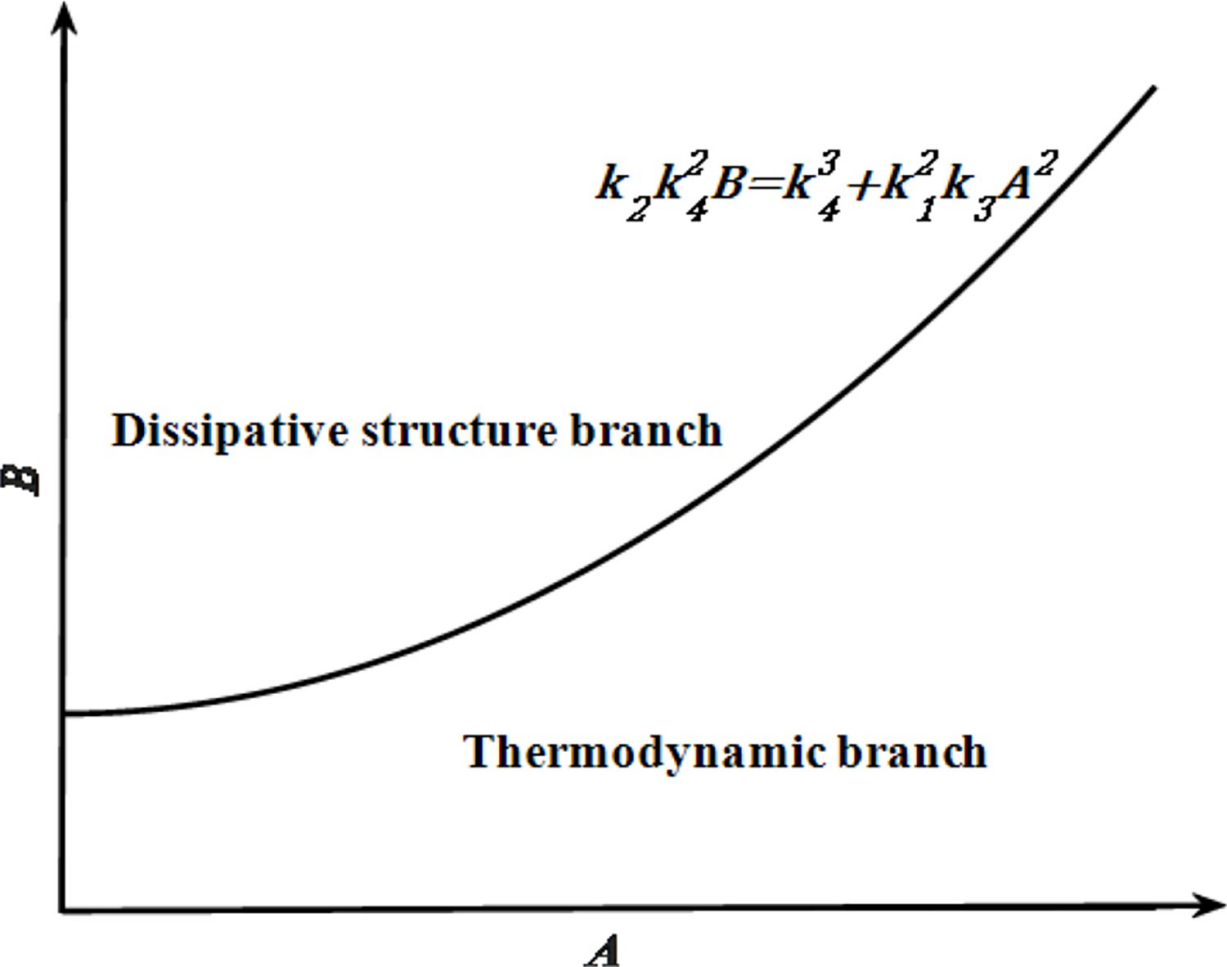
Schematic diagram of different branches in the parameter *A*−*B* plane.

As shown in Fig 3, $k_{2}k_{4}^{2}B=k_{4}^{3}+k_{1}^{2}k_{3}A^{2}$ is the bifurcation point where the system is in the bifurcation of the thermodynamic branch and dissipative structure branch. When $k_{2}k_{4}^{2}B<k_{4}^{3}+k_{1}^{2}k_{3}A^{2}$, the system is in the equilibrium state of the thermodynamic branch; when $k_{2}k_{4}^{2}B>k_{4}^{3}+k_{1}^{2}k_{3}A^{2}$, the system can form a dissipative structure and remain stable.

### Data processing

According to the principles of concise, scientific, systematic, and quantifiable indicators, the index system of *A* and *B* is constructed (see Table 2). For consistency and availability of data, this paper selects the relevant index data from 30 provinces and cities in China from 2015 to 2019 (Tibet is not included due to its interference with the overall data processing) and conducts an empirical analysis of the dissipative structure of SPRDSS in China. The original data was obtained from the National Bureau of Statistics, the China Macroeconomic Database, the EPS database, and the 2016–2020 "China Science and Technology Statistical Yearbook".

**Table 2:** The Brusselator model element index system.

Variables	Dimensionalities	Indexes
Financial input *A*	Public Services	Financial expenditure on General public services per capita C1
	Education	Financial expenditure on education per capita C2
	Science and Technology	Financial expenditure on science and technology per capita C3
	Culture, Sports, and Media	Financial expenditure on culture, sports, and media per capita C4
	Health Care	Financial expenditure on health care per capita C5
	Social Security	Financial expenditure on social security and employment per capita C6
Environment *B*	Economy	Regional GDP per capita C7
		Disposable income per capita C8
	Society	The population of urban and rural residents C9
		The proportion of urban population C10
	Technology	Internet penetration rate C11
		Cellphone penetration rate C12

To avoid the difference in data dimension among the indicators, we have to normalize the data with the below method.


$$\{\begin{array}{l} X_{ij}=\frac{x_{ij}-\underset{i}{\min}\{x_{ij}\}}{\underset{i}{\max}\{x_{ij}\}-\underset{i}{\min}\{x_{ij}\}},\;if\;x_{ij}\;is\;a\;positive\;indicator\\ X_{ij}=\frac{\underset{i}{\max}\{x_{ij}\}-x_{ij}}{\underset{i}{\max}\{x_{ij}\}-\underset{i}{\min}\{x_{ij}\}},\;if\;x_{ij}\;is\;negative\;indicator \end{array}$$ (10)

Where *X*_*ij*_ and *x*_*ij*_ refer to the normalized and original values of the *j*-th variable for the *i*-th object, respectively. And $\underset{i}{\max}{\{x}_{ij}\}$ and $\underset{i}{\min}{\{x}_{ij}\}$ refer to the maximum and minimum values of the *j*-th variable among the *i*-th objects, respectively.

After normalizing the indicators into [0,1], we used the Entropy Weight Method to calculate the weights of the indicators. The method proceeds in the following stages [38].

1. Calculating the entropy of the *j*-th variable is as follows.

$$E_{j}=-k\overset{n}{\underset{i=1}{\sum }}P_{ij}\ln P_{ij}$$ (11)

In which $P_{ij}=\frac{X_{ij}}{\sum_{i=1}^{n}X_{ij}},k=\frac{1}{\ln\left(n\right)}>0$, and *E*_*j*_>0.

2. Calculating the entropy weight of the *j*-th variable as follows.

$$w_{j}=\frac{1-E_{j}}{n-\sum_{j=1}^{n}E_{j}}$$ (12)

After calculating weights, the weighted sum method is used to calculate the values of *A* and *B*, as follows.


$$H_{i}=\overset{n}{\underset{j=1}{\sum }}w_{j}X_{ij}$$ (13)

Where *i* = *A*, *B*, and *H*_*A*_ and *H*_*B*_ refer to the values of *A* and *B*, respectively. Accordingly, the discriminant value $D\_value=k_{2}k_{4}^{2}B-k_{4}^{3}-k_{1}^{2}k_{3}A^{2}$ can be obtained.

## Results and discussions

Based on the above index system in Table 2, relevant original data were collected and then normalized. The values of financial inputs *A* and environment support *B* can be obtained by the Entropy Weighting Method, as shown in Table 3.

**Table 3:** The value of *A* and *B*.

Variables	*H*_*A*_					*H*_*B*_				
Provinces	2015	2016	2017	2018	2019	2015	2016	2017	2018	2019
BJ	0.9229	0.9123	0.9556	0.9789	0.9902	0.8665	0.8513	0.8382	0.8334	0.8383
TJ	0.5712	0.5764	0.4846	0.4660	0.4269	0.4816	0.4837	0.4861	0.4624	0.4546
HE	0.0412	0.0453	0.0538	0.0699	0.0749	0.2544	0.2701	0.2734	0.2648	0.2600
SX	0.1302	0.1030	0.1121	0.1347	0.1266	0.2359	0.2203	0.2282	0.2172	0.2170
NM	0.3514	0.2992	0.3164	0.2806	0.2621	0.2912	0.2884	0.2940	0.2885	0.2801
LN	0.1682	0.1414	0.1328	0.1522	0.1266	0.3915	0.3648	0.3563	0.3330	0.3326
JL	0.2186	0.2053	0.1999	0.1980	0.1717	0.2151	0.2180	0.2160	0.2065	0.1935
HL	0.1412	0.1174	0.1222	0.1293	0.1177	0.2073	0.2090	0.2043	0.1931	0.1900
SH	0.5910	0.7881	0.7915	0.7445	0.6723	0.7898	0.7897	0.7850	0.7911	0.7935
JS	0.2214	0.2257	0.2188	0.2215	0.2390	0.5224	0.5261	0.5315	0.5219	0.5161
ZJ	0.2182	0.2282	0.2252	0.2332	0.2863	0.5853	0.5668	0.5674	0.5633	0.5660
AH	0.0988	0.1377	0.1280	0.1278	0.1493	0.1737	0.1943	0.2123	0.2111	0.2004
FJ	0.1295	0.1247	0.1291	0.1234	0.1259	0.4696	0.4481	0.4453	0.4309	0.4393
JX	0.1373	0.1387	0.1581	0.1812	0.1987	0.1483	0.1711	0.1826	0.1872	0.1721
SD	0.0784	0.0825	0.0750	0.0751	0.0888	0.3735	0.3880	0.3944	0.3749	0.3565
HA	0.0376	0.0388	0.0440	0.0549	0.0681	0.2147	0.2313	0.2428	0.2398	0.2318
HB	0.1796	0.2022	0.1905	0.1772	0.1895	0.2691	0.2843	0.2909	0.2905	0.3119
HN	0.0992	0.1051	0.1141	0.1154	0.1179	0.1991	0.2214	0.2357	0.2322	0.2240
GD	0.1733	0.2441	0.2456	0.2496	0.2807	0.6322	0.6149	0.6214	0.6155	0.5992
GX	0.0951	0.0974	0.0952	0.0936	0.0938	0.1470	0.1531	0.1674	0.1666	0.1561
HI	0.2757	0.2606	0.2222	0.2730	0.3095	0.2194	0.2083	0.2025	0.1913	0.2005
CQ	0.1905	0.1759	0.1626	0.1680	0.1647	0.2693	0.2815	0.2960	0.2946	0.2849
SC	0.1106	0.1131	0.1104	0.1242	0.1289	0.2044	0.2217	0.2313	0.2440	0.2370
GZ	0.1752	0.1737	0.1776	0.1715	0.1894	0.0877	0.1041	0.1203	0.1349	0.1239
YN	0.1230	0.1361	0.1588	0.1550	0.1416	0.1110	0.1207	0.1272	0.1243	0.1254
SN	0.2146	0.1905	0.1911	0.1972	0.1789	0.2473	0.2477	0.2560	0.2606	0.2523
GS	0.2242	0.2057	0.1856	0.1911	0.1954	0.0717	0.0773	0.0884	0.0864	0.0794
QH	0.6494	0.5448	0.5620	0.5658	0.5698	0.1773	0.1615	0.1600	0.1615	0.1632
NX	0.3014	0.2973	0.2991	0.3003	0.2709	0.1888	0.1885	0.1961	0.1986	0.1817
XJ	0.3392	0.3086	0.2886	0.2739	0.2663	0.2018	0.1818	0.1810	0.1827	0.1790

According to Table 3, we can get *D*_*value* and determine whether SPRDSS forms a dissipative structure. The final results are shown in Table 4.

**Table 4:** Discriminant values of the dissipative structure of SPRDSS.

Provinces	2015		2016		2017		2018		2019	
	*D*_*value*	result	*D*_*value*	result	*D*_*value*	result	*D*_*value*	result	*D*_*value*	result
BJ	-0.0041	No	-0.0040	No	-0.0089	No	-0.0115	No	-0.0123	No
TJ	-0.0018	No	-0.0020	No	0.0030	Yes	0.0024	Yes	0.0037	Yes
HE	0.0002	Yes	0.0012	Yes	0.0013	Yes	0.0007	Yes	0.0003	Yes
SX	-0.0017	No	-0.0024	No	-0.0020	No	-0.0030	No	-0.0029	No
NM	-0.0036	No	-0.0021	No	-0.0023	No	-0.0015	No	-0.0016	No
LN	0.0074	Yes	0.0062	Yes	0.0058	Yes	0.0040	Yes	0.0044	Yes
JL	-0.0046	No	-0.0041	No	-0.0041	No	-0.0047	No	-0.0050	No
HL	-0.0037	No	-0.0033	No	-0.0036	No	-0.0044	No	-0.0044	No
SH	0.0163	Yes	0.0027	Yes	0.0021	Yes	0.0061	Yes	0.0114	Yes
JS	0.0146	Yes	0.0147	Yes	0.0152	Yes	0.0145	Yes	0.0138	Yes
ZJ	0.0186	Yes	0.0172	Yes	0.0173	Yes	0.0169	Yes	0.0156	Yes
AH	-0.0053	No	-0.0044	No	-0.0032	No	-0.0033	No	-0.0042	No
FJ	0.0129	Yes	0.0116	Yes	0.0114	Yes	0.0105	Yes	0.0110	Yes
JX	-0.0073	No	-0.0059	No	-0.0055	No	-0.0056	No	-0.0068	No
SD	0.0074	Yes	0.0083	Yes	0.0087	Yes	0.0075	Yes	0.0063	Yes
HA	-0.0023	No	-0.0012	No	-0.0005	No	-0.0008	No	-0.0014	No
HB	-0.0004	No	0.0001	Yes	0.0007	Yes	0.0010	Yes	0.0021	Yes
HN	-0.0037	No	-0.0023	No	-0.0015	No	-0.0018	No	-0.0023	No
GD	0.0224	Yes	0.0198	Yes	0.0202	Yes	0.0197	Yes	0.0179	Yes
GX	-0.0069	No	-0.0065	No	-0.0056	No	-0.0056	No	-0.0063	No
HI	-0.0057	No	-0.0060	No	-0.0054	No	-0.0074	No	-0.0079	No
CQ	-0.0006	No	0.0004	Yes	0.0016	Yes	0.0014	Yes	0.0008	Yes
SC	-0.0035	No	-0.0024	No	-0.0018	No	-0.0011	No	-0.0016	No
GZ	-0.0117	No	-0.0106	No	-0.0097	No	-0.0087	No	-0.0097	No
YN	-0.0094	No	-0.0090	No	-0.0089	No	-0.0091	No	-0.0088	No
SN	-0.0025	No	-0.0020	No	-0.0015	No	-0.0013	No	-0.0015	No
GS	-0.0137	No	-0.0129	No	-0.0118	No	-0.0121	No	-0.0126	No
QH	-0.0256	No	-0.0204	No	-0.0214	No	-0.0215	No	-0.0217	No
NX	-0.0084	No	-0.0083	No	-0.0078	No	-0.0077	No	-0.0079	No
XJ	-0.0088	No	-0.0090	No	-0.0085	No	-0.0080	No	-0.0080	No

**Fig 4:**
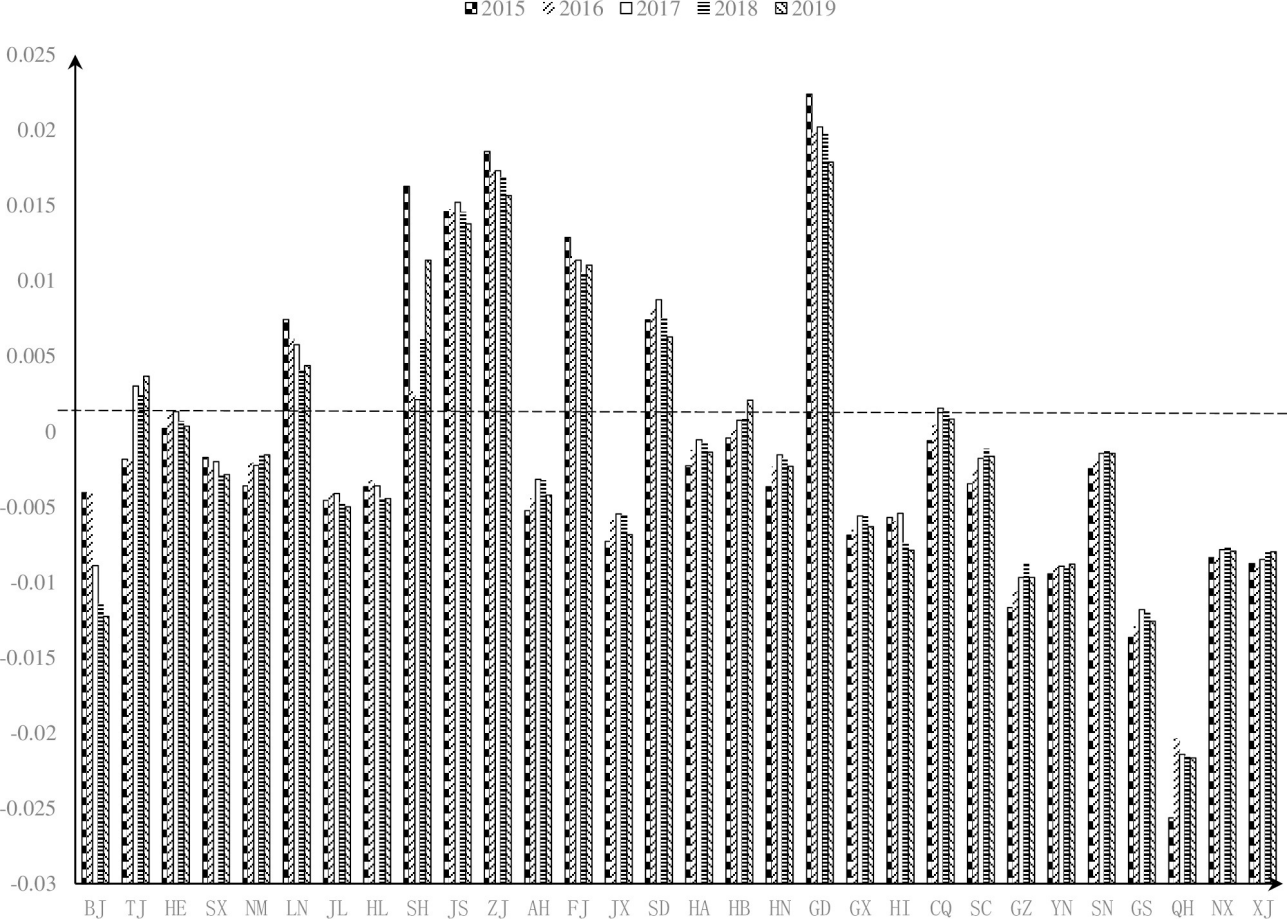
Dissipative structure discriminant value of digital sharing system of SPR in 2015–2019.

As shown in Fig 4, the quantity of SPRDSS with dissipative structure in China increased from 8 in 2015 to 11 in 2017, and it stayed unchanged in the following two years. Generally, there only are 36.7% of the provinces and cities in China whose SPRDSSs have formed the dissipative structure, and the rest of them are still in a thermodynamic branch. It can be explained that the proportion of capital investment and environmental support is not coordinated in most systems. This inconsistency can lead to a low sharing level and unbalanced regional socio-economic development. From 2015 to 2019, the discriminant values of most provinces and cities whose SPRDSSs have never formed the dissipative structure increased. In other words, these systems are gradually evolving into the dissipative structure branch. It indicates that with the in-depth implementation of policies such as new urbanization and rural revitalization, regional economic, social, and technological support has been strengthened, and the sharing level has been gradually improved. The higher the balanced degree of the regional development, the more resource sharing benefits. Their mutual promotion enables more and more SPRDSSs in China’s provinces and cities gradually evolved into the dissipative structure. In addition, our empirical results show that the dissipative structure of the system has not been changed for more than 4 years in 10 provinces and cities. These phenomena indirectly verify the dissipative structure theory that the system will not change into any new stable state until the thermodynamic branch state is destabilized, but only periodically with time. Therefore, when an SPRDSS in a certain province or city achieves the dissipative structure, it will not change in the next period until new fluctuations and mutations are formed.

**Fig 5:**
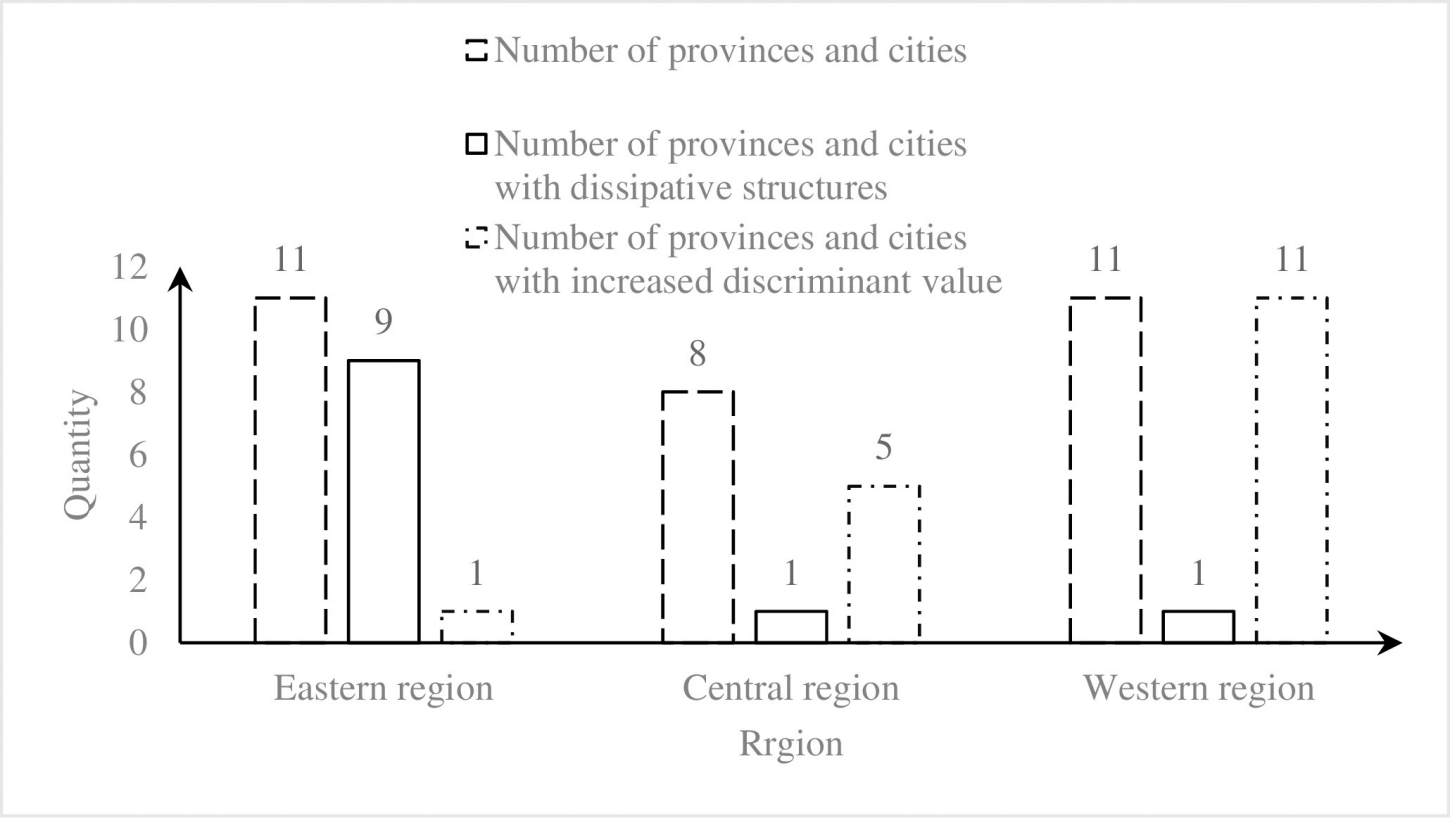
Comprehensive comparison of SPRDSS in 3-regions*. *The eastern region in China includes BJ, TJ, HE, SH, JS, ZJ, FJ, SD, GD, HN. The central region in China includes SX, AH, JX HA, HB, HN. The western region in China includes NM, GX, CQ, SC, GZ, YN, SN, GS, QH, NX, XJ.

The quantity and proportion of dissipative structure can be used to measure the global orderliness of SPRDSS, while the changing trend of the discriminant value can be used to measure the potential for improving the global orderliness of the system. As shown in Fig 5, from 2015 to 2019, there are nine provinces and cities in eastern China whose SPRDSSs have formed the dissipative structure, accounting for 81.8%. In central and western China, there is one province in each region whose SPRDSS reached the dissipative structure, accounting for 12.5% and 9.09% respectively. In central China, the discriminant values of the dissipative structure of 5 provinces and cities showed an oscillating growth trend, accounting for 62.5%. In western China, the discriminant values showed an increasing trend as a whole, but the values of some provinces show relatively slow growth.

In general, the orderliness of SPRDSS is greater in eastern China than in central China and is greater in central China than in western China. The potential for improving global orderliness is greater in western China than in central China and is greater in central China than in eastern China. The orderliness of SPRDSS is negatively correlated to its improvement potential. One possible explanation can be that the eastern coastal areas in China have better urbanization, superior socio-economic development, relatively higher living quality, excellent geographical location, and obvious population combined effect. In addition, advanced digital technology infrastructure and application level provide convenient technical environment support for digital sharing of SPR in eastern China. On the other hand, the industrial structure in central and western China is homogeneous. The economic development is relatively weaker. It is difficult to form excellent talent and technical environment support due to the restriction of geographic location. Hence, the orderliness of SPRDSS in eastern China is significantly higher than in central and western China. Another possible explanation is that, with the in-depth implementation of the new urbanization and rural revitalization, the under-developed central and western China has received a large inflow of technology, capital, talent, and matter. Their socio-economic conditions have been significantly improved, providing a solid physical foundation for the digital sharing of SPR. In addition, due to the low orderliness of SPRDSS, central and western China have more room for development, indicating a higher potential for improvement. In contrast, in the eastern coastal areas, the orderliness of SPRDSS is already high. The positive entropy of the system is constantly increasing in the process of operation, which decreases the potential for improving the orderliness of the system.

However, the SPRDSS in some provinces have not formed the dissipative structure, but their discriminant values are on a decreasing trend. The SPRDSSs in these provinces are evolving away from the dissipative structure branch. This may be due to the homogeneous industrial structure, the lack of economic development momentum, and the large population outflow in these areas. Specifically, these provinces rely on traditional manufacturing as the main supporting industry. There is great resistance to digital transformation in the high-quality development, which caused the slow regional economic growth and insufficient momentum. The serious population outflow makes it difficult to form a good talent, technology, and market environment and relatively weak demands for SPR.

## Conclusions and policy recommendations

Based on the panel data from 30 provinces and cities in China (except Tibet, Hong Kong, Macao, and Taiwan) from 2015 to 2019, this study conducts an empirical analysis of the formation conditions of the dissipative structure of SPRDSS. The results show that the SPRDSS has self-organizing features such as openness, fluctuation, far from equilibrium, and nonlinearity. It can enhance its internal order by exchanging material and energy such as capital, information, talent, and technology with the external environment, thus forming the dissipative structure. At present, there only 36.7% of the provinces and cities in China whose SPRDSSs have formed the dissipative structure. However, the discriminant values of the systems which have not formed the dissipative structure are gradually increased, which means that they are evolving into the dissipative structure. When the SPRDSS in a province or city forms a dissipative structure, the state will not change until new fluctuations and mutations occur. The global orderliness of SPRDSS is relatively high in eastern China and low in western China and it is distributed in a stepped pattern. The potential for improving global orderliness is greater in western China than in central China and is greater in central China than in eastern China. The global orderliness of SPRDSS in China shows a negative correlation to its potential for improvement.

Based on dissipative structure theory, this study uses the Brusselator model and its transformation to analyze the characteristics of the dissipative structure of SPRDSS in China and its formation conditions. It enriches and expands the theoretical research on digital sharing of SPR. In addition, it provides practical guidance for promoting digital sharing of SPR and high-quality development of regional economy from the following three perspectives.

Firstly, provinces and cities with the dissipative structure of SPRDSS should continue to increase financial investments into the system, introduce advanced technologies and talents, focus on product and technological innovation, and actively improve the application of ICTs in production and life. These will provide a stable and efficient environment for sharing and realize the sustainable stability of the dissipative structure. Secondly, for provinces and cities without the dissipative structure of SPRDSS, consensus should be made among the sharing entities of the system to lower the threshold of resource and information circulation and sharing and increase information disclosure to improve the circulation rate of resources. On one hand, they can strengthen the construction of sharing infrastructures and digital sharing platforms of SPR to guarantee the resources supply of the sharing system. On the other hand, they can promulgate sharing policies, collaborate within and between industries, enrich their industrial structures, attract the inflow of external talent, and actively create a favorable sharing cultural atmosphere and market environment. Last but not least, local governments are suggested to make sharing policies, according to the unique local socio-economic development conditions, and focus on the synergy and cooperation between SPRDSS in various areas. The eastern coastal areas should provide industrial support, talent, and technology transfer to the central and western regions. In this way, SPRPDSSs in the central and western regions can develop and upgrade faster and evolve to a more stable and advanced stage.

This paper comes with some limitations. Firstly, it only collects related data from 2015 to 2019, and new data are waiting for updating. Secondly, it fails to consider the intentions and motivations of the subjects using digital sharing. Subjects’ intentions to share can be influenced by many factors, such as policy institutions, digital technologies, and public demands. This issue needs to be addressed in the future studies.

## Supporting information

S1 Data Data in brief. (XLSX)
